# Influenza D Virus Infection in Herd of Cattle, Japan

**DOI:** 10.3201/eid2208.160362

**Published:** 2016-08

**Authors:** Shin Murakami, Maiko Endoh, Tomoya Kobayashi, Akiko Takenaka-Uema, James K. Chambers, Kazuyuki Uchida, Masugi Nishihara, Benjamin Hause, Taisuke Horimoto

**Affiliations:** University of Tokyo, Tokyo, Japan (S. Murakami, M. Endoh, T. Kobayashi, A. Takenaka-Uema, J.K. Chambers, K. Uchida, M. Nishihara, T. Horimoto);; Kansas State University, Manhattan, Kansas, USA (B. Hause)

**Keywords:** influenza, bovine, cattle, respiratory infections, infection, viruses, Japan

**To the Editor:** Although the provisionally named influenza D virus was first isolated as an influenza C–like virus from pigs with respiratory illness in Oklahoma in 2011 (*1*,*2*), epidemiologic analyses suggested that cattle are major reservoirs of this virus (*3*) and the virus is potentially involved in the bovine respiratory disease complex. The high rates of illness and death related to this disease in feedlot cattle are caused by multiple factors, including several viral and bacterial co-infections. Influenza D viruses were detected in cattle and pigs with respiratory diseases (and in some healthy cattle) in China (*4*), France (*5*), Italy (*6*), among other countries, indicating their wide global geographic distribution. Although the influenza D virus, like the human influenza C virus, is known to use 9-O-acetylated sialic acids as the cell receptor (*2*,*7*), its zoonotic potential is undefined because of limited research (*1*,*8*). We report influenza D virus infection in a herd of cattle in Japan.

To determine the presence of influenza D virus, on January 8, 2016, we used hemagglutination inhibition (HI) to test a convenience sample of 28 serum samples from healthy animals in a herd of female cattle in the Ibaraki Prefecture in central Japan. Two viruses with heterologous antigenicities, D/swine/Oklahoma/1334/2011 (D/OK) and D/bovine/Nebraska/9–5/2012 (D/NE) (*9*), were used for the assay with receptor-destroying enzyme (Denka: RDE II)–treated samples. Eight samples were positive for antibodies against both viruses, with HI titers of 1:80–1:640 for D/OK and with 2-fold or 4-fold lower HI titers (1:40–1:160) for D/NE in each sample, indicating previous infections in these cows, which ranged in age from ≈2 to 9 years. We also detected HI antibodies in serum samples from other cattle herds in several regions of Japan, although positivity rates varied (T. Horimoto, unpub. data). These data demonstrate the circulation of influenza D virus in Japan, as reported in other countries (*3*–*6*), emphasizing that the virus could be distributed worldwide.

Because 4 of the tested cows showed mild respiratory illness in January, we collected serum samples from the same 28 cows on February 3. At that time, only 1 cow still showed clinical signs; we collected a nasal swab sample from this cow. HI testing revealed that, of the 20 cows that had negative results in the first round of testing, 19 were positive for both D/OK and D/NE on the second test, which strongly confirms that influenza D virus infection had occurred and readily spread in this herd during January. However, most cows seemed to be subclinically infected with the virus. We cannot exclude the possibility of influenza D virus being a co-factor in causing respiratory illness because we did not evaluate the role of other viruses and bacteria in disease progression. HI titers (range 1:40–1:320) for D/NE were the same as or only 2 times lower than those for D/OK in all seroconversion samples, unlike the results for the seropositive samples in the first testing (Technical Appendix). This result indicates that the virus that spread in this herd in January might be different from the one that infected some cows before the second testing, which suggests the presence of multiple strains with different antigenicities in this area of Japan. No increase in HI titers was observed in the second testing of samples from the 8 cows that were antibody-positive in the first testing.

We used the nasal swab sample from the 1 cow with clinical signs for virus detection, although this cow possessed the HI antibody. Reverse transcription PCR that used specific primers (available from the authors by request) successfully amplified the full genome sequence (GenBank accession nos. LC128433 for D/bovine/Ibaraki/7768/2016), which was aligned to the influenza D virus sequence. However, we could not isolate infectious virus by using sensitive cells (*2*), which might be attributable to the delayed swab sample collection. Phylogenetic trees generated by using nucleotide sequences of individual segments from the Japan strain (95%–97% nucleotide identities with other strains) by maximum-likelihood analysis with ClustalW (http://www.clustal.org) and MEGA version 6.0 (*10*) indicated that this strain forms independent positions from strains isolated in other countries, although only the matrix segment was included in the same cluster as isolates from France and China (Figure). Although several unique amino acids of each protein exist in the strain from Japan, different from other isolates, their biologic characters are unknown. Among such residues, an amino acid at position 212 of hemagglutinin-esterase-fusion protein determined hemagglutinin antigenicity of the virus; lysine or arginine at this position resulted in heterologous antigenicities (*9*). The strain from Japan possessed serine at this position, identical to a strain from France (*5*), likely forming the third group of hemagglutinin antigenicity. Additionally, 1 putative N-glycosylation sequon was missing at positions 249–251 in hemagglutinin-esterase-fusion protein.

**Figure:**
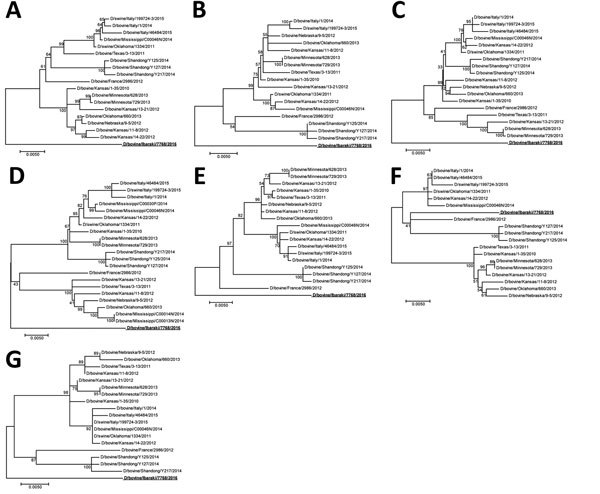
Phylogenetic trees of the 7 genomic segments of D/bovine/Ibaraki/7768/2016 (bold underline) at the nucleotide level. A) Polymerase basic protein 2; B) polymerase basic protein 1; C) polymerase protein 3; D) hemagglutinin-esterase-fusion protein; E) nucleoprotein; F) matrix protein; G) nonstructural protein. Maximum-likelihood analysis, in combination with 500 bootstrap replicates, was used to derive trees based on nucleotide sequences of the genome segments. Bootstrap values are shown above and to the left of the major nodes. Scale bars indicate the number of substitutions per site.

In summary, a cattle herd in Japan had influenza D virus infection. Although this study is a case report with a small number of samples, the observation shows a potential role for influenza D virus in the bovine respiratory disease complex.

Technical Appendix Detection of hemagglutination-inhibition antibodies to influenza D virus in a herd of cows in Ibaraki Prefecture, Japan
