# Supplementary material for: Influenza D Virus Infection in Herd of Cattle, Japan
Source: Emerg Infect Dis. 2016 Aug;22(8):1517–9. doi: 10.3201/eid2208.160362 (PMC4982187; doi:10.3201/eid2208.160362)
Supplement: Technical Appendix — Detection of hemagglutination-inhibition antibodies to influenza D virus in a herd of cows in Ibaraki Prefecture, Japan [file 16-0362-Techapp-s1.pdf]

Article DOI: http://dx.doi.org/10.3201/eid2208.160362

# Influenza D Virus Infection in Herd of Cattle, Japan

## Technical Appendix

**Technical Appendix Table.** Detection of HI antibodies to influenza D virus in a herd of cows in Ibaraki Prefecture, Japan*

| Cow no. | Age (as of 2016 Jan 8) | HI test titers 2016 Jan 8 D/OK | HI test titers 2016 Jan 8 D/NE | HI test titers 2016 Feb 3 D/OK | HI test titers 2016 Feb 3 D/NE | Clinical sign | Detection of virus |
|---|---|---|---|---|---|---|---|
| 7754 | 5 y 6 mo | <40 | <40 | 40 | 40 | | |
| 7761 | 4 y 5 mo | <40 | <40 | 80 | 80 | | |
| 7764 | 3 y 11 mo | <40 | <40 | 160 | 80 | | |
| 7765 | 3 y 10 mo | <40 | <40 | <40 | <40 | | |
| 7766 | 3 y 4 mo | <40 | <40 | 160 | 160 | | |
| 7767 | 3 y 4 mo | <40 | <40 | 80 | 80 | Respiratory illness | |
| 7768 | 3 y 4 mo | <40 | <40 | 160 | 160 | Respiratory illness | Nasal swab RT-PCR (+) |
| 7769 | 3 y 3 mo | <40 | <40 | 320 | 320 | Respiratory illness | |
| 7770 | 3 y 1 mo | <40 | <40 | 160 | 160 | Respiratory illness | |
| 7777 | 2 y 2 mo | <40 | <40 | 160 | 80 | | |
| 7779 | 2 y 1 mo | <40 | <40 | 160 | 80 | | |
| 7780 | 2 y 1 mo | <40 | <40 | 80 | 80 | | |
| 7781 | 2 y 0 mo | <40 | <40 | 80 | 40 | | |
| 7782 | 1 y 12 mo | <40 | <40 | 160 | 80 | | |
| 7784 | 1 y 7 mo | <40 | <40 | 160 | 80 | | |
| 7787 | 1 y 3 mo | <40 | <40 | 160 | 160 | | |
| 7788 | 1 y 0 mo | <40 | <40 | 80 | 40 | | |
| 7791 | 0 y 11 mo | <40 | <40 | 40 | 40 | | |
| 7795 | 0 y 8 mo | <40 | <40 | 320 | 160 | | |
| 7796 | 0 y 7 mo | <40 | <40 | 80 | 80 | | |
| 0472 | 7 y 9 mo | 80 | 40 | 160 | 80 | | |
| 0514 | 8 y 0 mo | 160 | 40 | 80 | 40 | | |
| 1336 | 6 y 11 mo | 80 | 40 | 160 | 80 | | |
| 1337 | 6 y 3 mo | 640 | 160 | 320 | 160 | | |
| 1412 | 7 y 10 mo | 160 | 40 | 80 | 40 | | |
| 5266 | 1 y 11 mo | 80 | 40 | 160 | 80 | | |
| 5270 | 1 y 10 mo | 80 | 40 | 40 | <40 | | |
| 8003 | 9 y 0 mo | 160 | 40 | 80 | 40 | | |

D/NE: D/bovine/Nebraska/9-5/2012; D/OK: D/swine/Oklahoma/1334/2011; HI, hemagglutination-inhibition; RT-PCR, reverse transcription PCR.
